# Soy-Based Infant Formula: Are Phyto-Oestrogens Still in Doubt?

**DOI:** 10.3389/fnut.2018.00110

**Published:** 2018-11-23

**Authors:** Ilaria Testa, Cristina Salvatori, Giuseppe Di Cara, Arianna Latini, Franco Frati, Stefania Troiani, Nicola Principi, Susanna Esposito

**Affiliations:** ^1^Pediatric Clinic, Department of Surgical and Biomedical Sciences, Università degli Studi di Perugia, Perugia, Italy; ^2^Neonatology and Neonatal Intensive Care Unit, Azienda Ospedaliera Santa Maria della Misericordia, Perugia, Italy

**Keywords:** hypothyroidism, infant nutrition, isoflavones, phyto-oestrogens, soy-based formulas

## Abstract

Although Scientific Societies have stated that there are very few indications for the use of soy-based formula (SF) in infant nutrition, their utilization rates have been repeatedly found to be higher than expected. It is likely that a significant role in this regard is played by the belief that the use of SF during infancy can reduce the risk of the development of several diseases later in life. Although no definitive data that can substantiate these claims have been collected, many people perceive soy consumption to confer significant health benefits and might also use soy for infant nutrition. However, not all the problems regarding safety of SF in infants have been definitively solved. Among risks, the potentially toxic role of the phyto-oestrogens contained in SF is not definitively established. *In vitro* and animal studies have raised suspicions that SF could have potentially negative effects on sexual development and reproductive function, neurobehavioral development, immune function, and thyroid function. Several studies in humans have aimed to assess whether the results of animal studies can be applied to humans and whether SF can be used in infants following the official recommendations. The results are somewhat conflicting. The aim of this narrative review is to discuss what is presently known regarding the impact of phyto-oestrogens in SF on early and late child development. PubMed was used to search for the studies published from January 1980 to June 2017 using the keywords: “soy,” “soy formula,” “child,” “phytoestrogens.” Analysis of the literature showed that a global evaluation of the impact of modern SFs on human development seems to suggest that their use is not associated with relevant abnormalities. Only children with congenital hypothyroidism need adequate monitoring of thyroid function.

## Introduction

Although several studies have shown that presently available soy-based formulas (SF) can allow for the normal growth and development of full-term infants (1–4), both the American Academy of Pediatrics (AAP) (5) and the European Society for Pediatric Gastroenterology, Hepatology, and Nutrition (ESPGHAN) (6) have stated that there are very few indications for the use of SF in infant nutrition. SF are not considered suitable for premature infant nutrition. Moreover, in full-term infants, galactosemia, and hereditary lactase deficiency are the only clinical conditions for which SF are considered the best solution for feeding infants. Finally, they can be used when a vegan diet is preferred. Other clinical conditions that were initially considered possible indications for SF use are presently preferentially treated with different nutritional approaches.

Although more expensive and less palatable than SF, extensively hydrolysed protein formulas are preferred by health authorities for infants with cow milk protein (CMP) allergy because in 10–14% of the cases, these patients also suffer from soy protein allergy (5, 6). Primary and post-gastroenteritis lactose intolerance can be treated with lactose-free CMF (5, 6). Moreover, patients with infant colic or supposed formula intolerance (spitting, vomiting, and fussiness) have no real benefit when treated with SF. Colicky behavior spontaneously reduces within a few months without nutritional modification, and perceived formula intolerance does not respond to changes in infant formula.

However, the utilization rates of SF have been repeatedly found to be higher than expected compared with the reported incidence of overmentioned indications for SF use. In Canada in 2005 (7) and in the USA in 2008 (5), it was reported that SF accounted for nearly 20 and 25% of the formula market, respectively. A recent evaluation of the consumption of different types of feeding among a nationally representative sample of 1,864 infants aged 0–12 months in the USA reported that among the 81% of infants who were fed formula or regular milk, 12% consumed SF (8). Reasons for these higher utilization rates of SF are not precisely defined. However, it is likely that, together with poor compliance with expert recommendations, a significant role in this regard is played by the belief that the use of SF during infancy can reduce the risk of the development of several diseases later in life. The administration of diets including high amounts of soy has been suggested for the prevention and treatment of cardiovascular diseases, type 2 diabetes, and osteoporosis in adults (9–15). Although no definitive data that can substantiate these claims have been collected (16), many people perceive soy consumption to confer significant health benefits and might also use soy for infant nutrition (17).

Moreover, not all the problems regarding safety of SF in infants have been definitively solved. In the past, it was shown that the high aluminum content of SF (500–2,500 mg/L vs. 15–400 mg/L and 4–65 mg/L in CMF and human milk [HM], respectively) could cause problems, particularly in premature infants and in subjects with reduced renal function (5). Along with neurological toxicity, aluminum competes with calcium for absorption, which can increase the risk of osteopenia, a condition that has been documented in 32% of 125 preterm infants SF fed, even if supplemented with calcium and vitamin D (18). Moreover, concerns have been raised for the presence of phytates (SF contains ~1.5% phytates), which may impair the absorption of minerals and trace elements (5). Both of these problems have been considered by experts and solved, at least in part, because modern SF contain very high amounts of phosphorus and calcium and are supplemented with iron and zinc.

In contrast, the potentially toxic role of the phyto-oestrogens contained in SF is not definitively established. *In vitro* and animal studies have raised suspicions that SF could have potentially negative effects on sexual development and reproductive function, neurobehavioral development, immune function, and thyroid function. Several studies in humans have aimed to assess whether the results of animal studies can be applied to humans and whether SF can be used in infants following the official recommendations. The results are somewhat conflicting.

This narrative review discusses what is presently known regarding the impact of phyto-oestrogens in SF on early and late child development. PubMed was used to search for the studies published from January 1980 to June 2017 using the keywords: “ soy,” “soy formula,” “child,” “phytoestrogens”. More than 1,200 articles were found, but only those published in English or providing data obtained from clinical studies with appropriate controls were included in the evaluation. This manuscript critically presents results published in 76 manuscripts.

## Phyto-estrogen charactheristics and biological activity

Phyto-oestrogens are plant compounds with oestrogenic activity. Those contained in SF are of the isoflavone class and include, in order of quantitative and biological importance, genistein, daidzein, and glycitein. All have a molecular structure quite similar to that of the human female hormone 17-β-oestradiol and, consequently, have oestrogenic activity, even if 1,000–10,000 times lower. They are present in very large amounts in SF, although with differences among commercial preparations (19). It has been calculated that the mean daily intake of isoflavones by an infant exclusively fed with one of the presently marketed SF can be as high as 11 mg/kg body weight, an amount significantly higher than that necessary to exert hormone-like effects in adults (20).

Over 94% of the phyto-oestrogens contained in SF are present as beta-glycosylated isoflavones, which are biologically inactive compounds that are very poorly absorbed. Activation occurs in the intestinal tract, where bacteria and intestinal beta-glucosidases remove glycosides from the ingested phyto-oestrogens, forming the so-called aglycones than can be easily absorbed (21). However, the number of active sugar-free compounds that are absorbed and enter circulation, exerting estrogen-like activity, varies from subject to subject according to the qualitative and quantitative composition of gut microbiota and the maturity of the gut mucosa. It is likely that those measures are lower in infants than in adults, as various perinatal and infant determinants, such as cesarean section delivery, gestational age, antibiotic treatment, or environmental factors, can affect the pattern of bacterial colonization and result in a different prevalence of metabolizing isoflavone bacteria (22). Moreover, before absorption, most aglycones are conjugated in the gut wall with glucuronic acid by uridine 5′-diphospho-glucuronosyltransferase or conjugate to sulfate by sulfotransferases, thus reducing the total number of active free aglycones that are absorbed (23). Finally, the liver plays a role in determining the concentration of active isoflavones in blood because a large portion of the absorbed aglycones are metabolized with the formation of compounds with low or negligible activity. This phenomenon explains why no more than 3% of the phyto-oestrogens detected in the blood of infants receiving SF are in active form (24). Despite these data, the concentrations of isoflavones in children receiving SF are significantly higher than those of children who consume HM or CMF, justifying the interest in the potentially negative hormonal effect of SF (25).

As they have a chemical structure similar to the hormone estrogen, sugar-free isoflavones can bind to both estrogen receptors (ER) α and β (26). However, its binding and transactivation are much weaker than that of oestrogens. Moreover, binding occurs preferentially to ERβ, in contrast to estrogen, which binds to and transactivates ERα and ERβ equally. This difference can have relevant clinical importance because the two ERs have different tissue distributions and functions and, when activated, can have different and sometimes even opposite physiological effects. ERα can be mainly detected in the mammary glands, uterus, ovaries (thecal cells), bone, testes, and epididymis, prostate (stroma), liver, and adipose tissue. By contrast, ERβ is found mainly in the prostate (epithelium), bladder, ovaries (granulosa cells), colon, adipose tissue, and immune system. Both subtypes are markedly expressed in the cardiovascular and central nervous systems. The preference of isoflavones for ERβ is the primary reason that isoflavones are seen as capable of having tissue-selective effects (27). However, phyto-oestrogens have several other ER-independent functions, including the alteration of epigenetic marks and the inhibition of oestradiol, which can play a role, at least theoretically, in conditioning normal sexual development and reproductive function (28). Finally, phyto-oestrogens affect T3 and T4 synthesis by inhibiting thyroid peroxidase (TPO). Moreover, they interfere with the absorption of administered thyroid hormone (29, 30).

## Effects of phyto-oestrogens in animals

Reports of the potential relationship between phyto-oestrogens and abnormalities in the reproductive health of animals date back to more than 50 years ago, when it was evidenced that sheep and cows that grazed on red clover, one of the richest sources of isoflavones, were infertile (31, 32). Similar findings were reported in captive cheetahs eating soy-based diets (33). As the reduction of phyto-estrogen intake restored fertility, it was concluded that phyto-oestrogens had a negative role on animal reproduction (34). Starting from these premises, in the following years, several experiments were carried out to study the early and late effects of isoflavones on developing animals. With some exceptions (35–37), the results of these studies have suggested a potentially negative role of the early administration of isoflavones, although with differences among rodents and non-human primates and between short-term and long-term evaluation.

The ingestion of genistein by female rats during pregnancy and the lactation period in doses that included the estimated range of infant human isoflavone exposures through SF was found to be associated with low birth weight and with a large series of developmental abnormalities in puppies sacrificed at 50 days of life. The effects were strictly dose- and sex-related. In male pups, particularly those receiving the highest dose, a decreased ventral prostate weight and a trend toward higher pituitary gland to body weight ratios was found. Ductal/alveolar hyperplasia of the mammary glands, aberrant or delayed spermatogenesis in the seminiferous tubules, a deficit of sperm in the epididymis, and an increase in the incidence and/or severity of renal tubal mineralization were also evidenced. In female pups, similar abnormalities in the pituitary gland and in mammary glands were demonstrated, together with abnormal cellular maturation and abnormal ovarian antral follicles (38). Moreover, it was shown that rats that were exposed to isoflavones during fetal life and adolescence had a sexual development quite different from that of animals fed with a diet lacking isoflavones (39). Exposure to isoflavones was associated with a precocious vaginal opening and, later in life, with more frequent irregular oestrus, higher levels of follicle stimulating hormone (FSH) and greater uterine epithelial height. In marmoset monkeys, the intake of an amount of isoflavones 40–87% of that reported in 4-months human infants exclusively fed with SF resulted in a consistently lower mean testosterone level compared to animals fed with standard CMF. At day 35–45, paired comparisons revealed 53–70% lower levels in 11 of 13 co-twins fed with SF (*p* = 0.004) (40). In the same animals, exclusive feeding with SF for the first 5–6 weeks of life was not associated at 120–138 weeks of age with any abnormality of weight, puberty initiation, and progression, prostate, seminal vesicles, pituitary, thymus and spleen weight, and penis length. Six out of 7 studied animals were fertile. However, testis weight (*p* = 0.041), and Sertoli (*p* = 0.025) and Leydig cell (*p* = 0.026) numbers per testis were consistently increased (40). Moreover, the increase in Leydig cell numbers was strictly inversely related to testosterone levels. These findings seem to indicate that in primates the intake of isoflavones in infancy has no effect on the timing or progression of puberty, on fertility and on immune system development, as suggested by the normal thymus and spleen weight. However, the increase in testis weight and in the number of Leydig cells may indicate a compensated Leydig cell failure, possibly a consequence of the early influence of isoflavones (41).

Along with alterations of sex organ development, isoflavones given to rats have been associated with abnormal brain maturation and function and, in contrast to what has been previously highlighted, structural abnormalities of the immune system. Faber et al. reported that exposure of neonatal female rats to high doses of genistein alters post-pubertal pituitary response to gonadotropin-releasing hormone and increases the volume of the sexually dimorphic nucleus of the preoptic area, leading to levels normally encountered in male animals (42). Moreover, phyto-oestrogens have been associated with increased seizure susceptibility (43). Regarding the immune system, in female mice, genistein subcutaneous injections producing serum levels lower that those found in infants fed with SBF resulted in marked thymic atrophy associated with impaired maturation of helper T cell lineage and systemic lymphopenia (44).

Finally, it has been shown that isoflavones inhibit the activity of TPO in rats (45, 46). The effects are strictly dose-dependent. This finding explains why in some experimental studies, thyroid hormone levels (T3, T4, TSH) in serum, thyroid weights, and histopathology were found to be similar in animals receiving genistein and in untreated controls, suggesting that the residual TPO activity was sufficient to maintain thyroid homeostasis in the absence of additional perturbations.

## Effect of soy-based formulas (sbf) in children

Starting from the results of the experimental studies, it could be supposed that SF-fed children, particularly the youngest, can have significant clinical problems. Fortunately, the analysis of the data that have measured short- and long-term impact of early introduction of soy in infant nutrition are far less pessimistic. In most of the cases, poor or no negative effect has been demonstrated. Presently, there is no conclusive evidence that SF can significant affect human development, reproduction and endocrine function. Only hypothyroid children can have significant problems. Several factors can explain the differences between experimental and human studies. Animal studies typically examine the effects of isolated compounds, mainly genistein, whereas SF used for infant nutrition contains several other components, including different aglycones. Moreover, doses, route and duration of administration have frequently been significantly different from those regarding SF use in infants. Finally, in all the commonly used experimental animals, including monkeys, the metabolism of isoflavones is very different from that demonstrated in humans, suggesting that the same dose of isoflavones can lead to different serum levels of aglycones.

### Reproductive system

The safety of SF in relation to reproductive and endocrine functions was evaluated by Vandenplas et al. (1). The cross-sectional, case–control, and cohort studies and clinical trials published until mid-2013, in which the sexual development of SBF-fed children was compared with that of children receiving HM and/or other infant formulas, were analyzed. Only 4 studies were identified, and their analysis led the authors to conclude that, although some differences between groups could be detected, the long-term effects of isoflavones on important reproductive functions in human beings were marginal and clinically irrelevant. In particular, it was shown that children exposed to isoflavones during fetal life or early infancy could have a modest increase in breast tissue at 2 years of age, an early menarche, a longer menstrual period and more significant menstrual discomfort. However, an increase in breast tissue was considered a transient phenomenon because it was only the consequence of a slower waning of the infantile breast (47, 48), the advance of menarche was limited to very few months (age of menarche 12.4 years in SF-fed girls vs. 12.8 years in the entire studied population) (47, 49), the prolongation of bleeding during menstrual periods was only 9 h, and discomfort was only slightly increased (50).

The long-term safety of SF feeding was also supported by the evidence that, when a complete evaluation of reproductive system characteristics was performed in the analyzed studies, more severe reproductive problems were excluded. For example, in the study by Strom et al. in which only subtle menstrual problems were evidenced, other signs or symptoms of SF toxicity on the reproductive system, including early thelarche and puberty, modification of cycle length, severity of menstrual flow, irregular or missed menstrual periods, breast tenderness, and infertility, were not detected (50). These findings seem to suggest that the evidence of a potential relationship between exposure to isoflavones in the first months of life and the development of permanent modifications of reproductive system function was a chance occurrence. Moreover, this evidence seems to minimize the clinical relevance of studies that have found that infant girls fed SF had vaginal cytological changes consistent with estrogen exposure (51), including higher DNA methylation at a specific gene locus. Harlig et al. examined the effect of neonatal exposure to genistein on gene-specific mRNA levels in vaginal tissue and found that DNA methylation at PRR5 L was high at birth and fell rapidly in the 2 months after birth only in CMF-fed children because of the rapidly falling exposure to maternal oestrogens (52, 53). In contrast, SF-fed children maintained higher methylation levels over time, and this finding suggested persistent oestrogenic stimulation by isoflavones (52).

On the other hand, most of the studies not included in the Vandenplas et al. analysis did not find significant toxic effects of isoflavones on the reproductive system (1). Breast bud, uterine, ovarian, prostate, and testicular volumes were measured in boys and girls fed with SF and compared with those of matched subjects receiving HM or CMF. These groups were compared at 4 months (54) and 5 years of age (55). In both cases, no evidence was detected that feeding SF could exert any short- and/or long-term oestrogenic effect on reproductive organs. A recent nested case-control study enrolling children aged 7.8–10.5 years whose eating habits were prospectively followed from birth until 3 years did not show any association between puberty and infantile nutrition (56). The lack of any influence of SBF on the sexual development of male children was confirmed by studies that showed no risk of hypospadias (57) or gynaecomastia (58), being equally common in SF-fed male infants than in subjects receiving different types of milk. Finally, the reported increased risk of uterine fibroids in women who had been SF-fed during infancy is debatable. Statistical analysis does not support the conclusion of the study by D'Aloisio et al. (59). Although the authors have calculated a risk ratio of 1.25, the *p*-value was not significant because of a 95% confidence interval of 0.97–1.61.

### Central nervous system

As brain electrical or electroencephalographic (EEG) activity in humans has been linked to the maturation of brain (60, 61) and behavioral and cognitive function (62), a number of studies including electrophysiological evaluations have been performed in infants receiving different types of milk to evaluate whether diet could have an influence on neurologic development. Data regarding SBF were generally comforting, as the measure of brain development through the evaluation of electrical activity revealed that SF had effects that were similar to those of CMF and, at least in some cases, not different from those of HM. All these findings suggest that SF adequately support brain development and function during the first postnatal periods and that isoflavones have no negative effect. Li et al. compared behavioral development and cortical responses to speech sounds in infants fed either HM or CMF or SF at 3 and 6 months of age (63). No diet-related differences were found, as event-related potential measures were generally similar, and behavioral measures were within normal ranges in all the groups. Pivik et al. studied temporal and frontal brain region responses associated with the processing and discrimination of speech sounds in infants receiving either HM, CMF, or SF during the first 6 months of life (64). All groups showed significantly greater response amplitudes to the speech sounds across sites at 3 months and frontally at 6 months.

Greater syllable discrimination was evidenced at 6 months in HM-fed infants, whereas no difference was evidenced in formula-fed infants, independent of the type of milk. Similar results were obtained when diet-related differences in EEG characteristics among differently fed infants during the first year of life were evaluated (65).

Moreover, evaluation of the long-term impact of SF on central nervous system function highlights that, even if slight differences in the electrophysiological maturation of the central nervous system truly exist, they have little or no relevance to long-term intellectual development. A recent study that evaluated the developmental status (mental, motor, and language) of HM-, MCF- or SF-fed infants during the first year of life showed that all the scores on developmental testing were within established normal ranges independent of diet, although scores were slightly higher in HM-fed than in MCF- and SF-fed children (66). Moreover, in a study carried out in 9- to 10-year-old children who were fed either SF or HM during their first year of life, differences in intelligence quotient, behavioral problems, learning impairment, and emotional problems were not found (67). Similar results were obtained when adults aged 20–34 years were examined. The percentage of men or women who achieved some level of school education was the same in SF- or CMF-fed subjects (50).

### Immune system

Reports published ~30 years ago have suggested that SF may interfere with immunization processes. The immune response to the polio, diphtheria, tetanus, pertussis (68), and rotavirus (69) vaccines was found to be lower in SF-fed infants than in controls. However, more recent studies with SF containing higher levels of proteins and fewer non-digestible carbohydrates did not confirm these findings, suggesting that SF do not compromise immune status. Ostrom et al. measured at 6, 7, and 12 months of age the antigen-specific immune responses to *Haemophilus influenzae* type b polysaccharide, diphtheria toxoid, tetanus toxoid, and oral poliovirus of children who were immunized at 2, 4, and 6 months of age (70). Moreover, the incidence of infections during the study period was recorded. Infants fed SF showed similar immune responses to infants fed HM for at least the first 2 months of life (70). Serum IgG and IgA levels among feeding groups were not substantially different among the three time points. Morbidity throughout the first year of life was also similar, as the incidence of episodes of physician-reported otitis media and parent-reported diarrhea was independent of diet.

Finally, analysis of activation and maturation of B, T, and NK lymphocytes did not reveal any consistent differences in immune status, maturation, or level of immunocompetence between infants fed SBF and controls (71).

### Thyroid function

A relevant number of studies have evaluated relationships between soy intake and thyroid function in adults, mainly pre-menopausal and post-menopausal women. No clinically significant effects in healthy adults were found. In contrast, as isoflavones interfere with the absorption of administered thyroid hormone, diets including a high intake of isoflavones, as may occur in vegetarian diets, may lead to overt hypothyroidism patients with subclinical disease (72). Increased replacement doses may be needed in these subjects (73).

There are very few studies in children, and conclusions regarding the impact of SF on thyroid function during development are mainly based on case reports. Most of these reports were published in the 1960s (74, 75), before the introduction of iodine-supplemented SF (5). However, iodine supplementation can be inadequate in children with congenital hypothyroidism treated with conventional hormone replacement. Children receiving apparently adequate replacement doses may develop overt hypothyroidism when fed with SF (76, 77). The need for greater replacement doses is also supported by evidence that feeding SF to infants with congenital hypothyroidism leads to a prolonged increase in TSH. Conrad et al. studied the role of diet in conditioning thyroid function in children with congenital hypothyroidism and found that SF-fed patients were significantly different from controls on the following characteristics: time to TSH normalization, first TSH on treatment, percentage with increased TSH at 4 months of age, percentage with increased TSH throughout the first year of life, and overall trend of TSH at each visit (78). These findings led to the conclusion that although SBF-fed infants with congenital hypothyroidism receive normal replacement therapy, they still need close monitoring of free thyroxine and TSH measurements and may need increased levothyroxine doses to achieve normal thyroid function tests.

## Conclusions

A global evaluation of the impact of modern SFs on human development seems to suggest that their use is not associated with relevant abnormalities. The negative influence of isoflavones, which has been repeatedly demonstrated in developing animals, has not been evidenced with the same relevance in humans. Only children with congenital hypothyroidism can have problems and require remodulation of thyroid hormone replacement doses. It is highly likely that the suggestion of scientific societies for use of SF in infants, although they go back to several years ago (5, 6) can be maintained However, this does not mean that the potential harmful effects of soy isoflavones on child development can be definitively excluded. Isoflavones are transferred to the fetus through placenta (79) and are excreted in HM (20). The risks of children born to or breastfed by vegan mother are not established. Moreover, as absorption, distribution, metabolism, and excretion of isoflavones can significantly vary, it is possible that in some cases more pronounced effects can be manifest. Further studies are needed to evaluate the problem.

## Author contributions

IT and CS wrote the first draft of the manuscript. GD, FF, and ST revised the text. AL gave a support in the literature review. NP and SE critically revised the text and made substantial scientific contributions. All the authors approved the final version of the manuscript.

### Conflict of interest statement

The authors declare that the research was conducted in the absence of any commercial or financial relationships that could be construed as a potential conflict of interest.
